# Mechanism of traumatic heterotopic ossification: In search of injury‐induced osteogenic factors

**DOI:** 10.1111/jcmm.15735

**Published:** 2020-08-27

**Authors:** La Li, Rocky S. Tuan

**Affiliations:** ^1^ Center for Cellular and Molecular Engineering Department of Orthopaedic Surgery University of Pittsburgh School of Medicine Pittsburgh PA USA; ^2^ Institute for Tissue Engineering and Regenerative Medicine The Chinese University of Hong Kong Hong Kong SAR China

**Keywords:** bone morphogenetic protein, dystrophic calcification, glucocorticoid, heterotopic ossification, muscle injury, transforming growth factor‐β1

## Abstract

Heterotopic ossification (HO) is a pathological condition of abnormal bone formation in soft tissue. Three factors have been proposed as required to induce HO: (a) osteogenic precursor cells, (b) osteoinductive agents and (c) an osteoconductive environment. Since Urist's landmark discovery of bone induction in skeletal muscle tissue by demineralized bone matrix, it is generally believed that skeletal muscle itself is a conductive environment for osteogenesis and that resident progenitor cells in skeletal muscle are capable of differentiating into osteoblast to form bone. However, little is known about the naturally occurring osteoinductive agents that triggered this osteogenic response in the first place. This article provides a review of the emerging findings regarding distinct types of HO to summarize the current understanding of HO mechanisms, with special attention to the osteogenic factors that are induced following injury. Specifically, we hypothesize that muscle injury‐induced up‐regulation of local bone morphogenetic protein‐7 (BMP‐7) level, combined with glucocorticoid excess‐induced down‐regulation of circulating transforming growth factor‐β1 (TGF‐β1) level, could be an important causative mechanism of traumatic HO formation.

## 1 INTRODUCTION

Heterotopic ossification (HO) is a pathological condition of abnormal bone formation in soft tissue.^1^ Due to the abnormal mechanical effect of hard tissue present inside soft tissue, HO usually causes pain and restricted range of motion.^2^ HO can be generally divided into two broad categories: hereditary and acquired. Hereditary HO, also known as fibrodysplasia ossificans progressiva (FOP), is a rare autosomal dominant disease resulting from activin A receptor type I (*ACVR1*) gene mutation.^3^ Acquired HO typically follows central nervous system (CNS) injury or direct musculoskeletal trauma.^4^ However, the mechanism underlying acquired HO is still unclear. Although different types of HO do not utilize identical mechanistic pathways of pathogenesis, muscle injury appears to be a unifying feature for all types of HO.^5^ In FOP patients, low levels of muscular trauma following childhood immunizations or play‐related falls can result in HO.^6^ In neurogenic HO, microtrauma to muscles resulting from forced passive movements following a period of immobilization will lead to an increased risk of HO.^7^ In traumatic HO, more severe levels of muscular trauma, as in cases of arthroplasty and blast trauma, can lead to an HO incidence of 20% and 64.6%, respectively.^8^, ^9^ However, the precise mechanism by which muscle injury facilitates HO formation is still largely unknown.

Chalmers et al proposed that three factors were required to induce HO: (a) osteogenic precursor cells, (b) osteoinductive agents and (c) an osteoconductive environment.^10^ Since Urist's landmark discovery of bone induction in skeletal muscle tissue by demineralized bone matrix (bone morphogenetic protein [BMP] was later shown to be the active biological factor),^11^, ^12^ it is generally believed that skeletal muscle itself is a conductive environment for osteogenesis and that resident progenitor cells in skeletal muscle are capable of differentiating into osteoblast to form bone.^13^ Although it is possible that once osteogenic differentiation is initiated in the progenitor cells, they will exhibit self‐sustaining production of osteoinductive factors for continuous bone formation, little is known about the naturally occurring osteoinductive agents that triggered this osteogenic response in the first place. Muscle injury usually involves the initiation of an inflammatory response, and inflammatory cells are known to be the source of cytokines, chemokines and growth factors.^14^ It is thus likely that the progenitor cells respond to the osteoinductive signals produced by inflammatory cells to initiate the bone formation process. However, this does not explain why inflammation alone does not induce HO. Therefore, other factors must work in concert with the osteoinductive signals to allow for the initiation of osteogenesis. This article will provide a review of the emerging findings regarding distinct types of HO to summarize the current understanding of HO mechanisms, with special attention to the osteogenic factors that are induced following injury.

## 2 GENETIC HO

The most common form of hereditary HO is FOP. Progressive osseous heteroplasia (POH) is excluded from this review given that it progresses from deep layers of skin to skeletal muscle without an inflammation component in disease pathogenesis^15^, ^16^ and is thus likely to be aetiologically different from typical HO. FOP is a life‐threatening disease and has an incidence of 1 in 2 million individuals worldwide.^6^ Patients were born normal, only with congenital malformation of the hallux or big toe.^6^ The extraskeletal ossification begins in childhood following flare‐ups (painful soft tissue swelling), initiated by mild bodily trauma, such as childhood immunizations or play‐related falls.^6^ Progressive HO first forms around the trunk in affected patients and then proceeds to the whole body.^16^ Ectopic bone eventually spans the joints, which renders movements impossible.^16^ Most patients die of thoracic insufficiency syndrome before the age of around forty.^6^ FOP is caused by a point mutation in the *ACVR1* gene, which results in an amino acid change in codon 206 (R206H) within the glycine‐serine (GS) activation domain of a BMP type I receptor, activin receptor‐like kinase‐2 (ALK2).^3^ The consequence of this R206H mutation is constitutive activation of BMP signalling, although the exact mechanism concerning how this mutation perturbs BMP signalling is not totally clear. It is thought to be due to the altered binding of an inhibitory protein FKBP12 to the GS domain that leads to constitutive activation of ALK2.^3^

In addition to the genetic aspect of the disease aetiology, other interesting phenomena have been observed in FOP patients, in ex vivo study of FOP patient samples as well as in FOP animal models. First, given that FOP patients are born normal and HO is only initiated following flare‐ups caused by minor trauma or inflammation, immunological trigger for HO pathogenesis has been implicated.^17^, ^18^ It is actually well known that anti‐inflammatory drug treatment prevents HO formation, as high‐dose corticosteroids treatment started within the first 24 hours following injury has been used as prophylaxis against HO formation.^6^ In FOP animal models, suppression of inflammation and depletion of inflammatory cells typically inhibit HO formation.^19^, ^20^, ^21^ Recent studies have demonstrated that FOP connective tissue progenitor cells respond to inflammatory signals through toll‐like receptor (TLR) and ECSIT (Evolutionarily Conserved Signaling Intermediate in Toll Pathway) links TLR to BMP signalling.^17^, ^22^ This finding suggests a possible link between the innate immune system and dysregulated BMP signalling in tissue progenitor cells. Secondly, an unexpected finding was that activin A, usually acting as a BMP signalling pathway inhibitory ligand, can activate mutated ALK2 and its downstream Smad1/5 signalling pathway.^23^, ^24^ Studies have further demonstrated that R206H ALK2 mutation‐driven HO is a ligand‐dependent process, since BMP and activin ligand blockers (ACVR2A‐Fc and ACVR2B‐Fc) largely prevented HO from happening in the conditional *ACVR1* R206H knock‐in mice.^23^ Moreover, activin A treatment initiated HO formation, and treatment with anti‐activin A antibody blocked HO formation in the conditional *ACVR1* R206H knock‐in mice.^23^ Therefore, activin A is an obligate ligand in FOP, which may account for the dependence of HO on inflammation since activin plays a key role in the immune system.^18^

Cellular mechanistic studies have demonstrated that targeting *ACVR1* R206H to Tie‐2 promoter‐driven cre and PDGFRα promoter‐driven cre resulted in spontaneous and injury‐induced HO in the transgenic mice, while targeting *ACVR1* R206H to MyoD‐cre and VE‐cadherin‐cre did not, suggesting the fibro/adipogenic progenitors (FAPs) origin of the HO lesions.^25^ Activin A treatment also initiated HO formation in this FAP‐driven HO mouse model, and treatment with anti‐activin A antibody blocked HO formation in this model as well.^25^ Based on these findings, a working model for FOP mechanism could be that *ACVR1* R206H FAPs respond to injury‐induced activin A expression to form HO. Therefore, activin A could be the osteogenic factor for genetic HO. However, the question that remains to be answered is: when there is no mutated ALK2 to transduce the activin A signal as in acquired HO following CNS injury or direct musculoskeletal trauma, what are the injury‐induced osteogenic factors that account for the aberrant osteogenesis in FAPs?

## 3 NEUROGENIC HO

Neurogenic HO (NHO) is defined as HO following spinal cord injury (SCI) and traumatic brain injury (TBI).^26^ The incidence for NHO following CNS injury has been reported to be 1 in 5 patients.^27^ Ectopic bone formation begins within 2 months after neurologic injury and usually forms around large joints.^28^ It has also been reported that many patients with head injury have accelerated fracture healing, which has been considered as a variant of NHO.^29^ In some circumstances, injury to peripheral nerve could also compromise CNS integrity.^30^ NHO animal studies have demonstrated that peripheral denervation increased HO volume on the original SCI NHO model and peripheral denervation alone without SCI could also induce HO in about 50% of mice with local muscular injury.^31^ Risk factors for NHO include artificial ventilation, immobilization and muscular spasticity.^26^ Specifically, artificial ventilation alters blood homeostasis of electrolytes and acid‐base balance, both of which will affect osteogenesis.^29^ Immobilization and muscular spasticity have been suggested as a cause of increased risk of muscle tears from active or passive movement.^7^ However, NHO animal studies have demonstrated that blocking neuromuscular junction (NMJ) by botulinum toxin A (BTA) as a clinical approach to reduce spasticity actually enhances HO formation in the original SCI NHO model.^32^ This could possibly be explained by the fact that BTA injection equals to a denervation model, and peripheral denervation has been previously shown to enhance NHO.^31^

The aetiology of NHO is incompletely understood. A general hypothesis for NHO pathogenesis involves the release of osteogenic humoral factors following the breakdown of blood‐brain barrier.^33^, ^34^ In vitro studies support this notion by demonstrating that the cerebrospinal fluid (CSF) and serum from patients with severe TBI have an osteoinductive effect and are able to stimulate osteoblastic cell proliferation.^35^, ^36^, ^37^ Peripheral nerve system may also participate in NHO through neuroinflammation, which refers to a process in which CNS injury triggers inflammatory reactions in peripheral tissue through the release of substances from the peripheral terminals of peptidergic, sensory nerve fibres.^38^ Indeed, the neural‐inflammatory factor, substance P (SP), has been found to be dramatically increased in NHO lesions^39^ and in the plasma of NHO patients.^40^ Animal studies also showed that in mice lacking functional sensory neurons or in mice with null mutation of SP gene, BMP‐induced HO was dramatically inhibited.^39^, ^41^

Injury‐induced inflammation also exerts paramount influences on NHO pathogenesis. Depletion of macrophages by clodronate‐loaded liposomes reduced the size of NHO by 90% in a combined thoracic spinal cord transection plus local muscular trauma NHO mouse model.^40^ In addition, conditioned medium derived from activated CD14+ monocytes/macrophages isolated from NHO lesion strongly stimulated the mineralization of muscle‐derived stromal cells (MDSCs) isolated from the same site, suggesting the participation of inflammatory cells in NHO pathogenesis.^42^ Recently, oncostatin M (OSM) has been found to be the coupling factor linking macrophages and MDSCs.^42^ Findings that support this notion are as follows. Plasma OSM level significantly increased in NHO patients compared with healthy controls.^42^ Treatment with OSM neutralizing antibody greatly reduced the osteogenesis stimulating effect of conditioned medium derived from activated CD14+ monocytes/macrophages isolated from NHO lesion.^42^ Mice that are deficient for OSM receptor as well as mice treated with JAK1/2 tyrosine kinase inhibitor ruxolitinib had significantly reduced HO volume compared with wild‐type controls after SCI and local muscular trauma.^42^, ^43^ However, since deficiency of OSM receptor and inhibition of JAK/STAT3 signalling pathway did not achieve the same effect with depletion of macrophages, other injury‐induced osteogenic factors linking macrophages with MDSCs should also exist and have yet to be explored.

## 4 TRAUMATIC HO

Traumatic HO is typically considered as HO triggered solely by injury, most often following extensive soft tissue injury.^44^ The rate for HO happening following distal humerus fractures is about 8.6%, and elbow fractures about 5.5%‐18.8%.^45^ The rate can reach up to 90% following acetabular fractures and total hip arthroplasty (THA).^46^ HO that occurs subsequent to thermal injury ranges from 0.2% to 4%, with elbow being the most affected site.^47^ The highest rate of HO happens in combat‐related blast injuries, the prevalence of which is reported to be 64.6% in high‐energy wartime extremity wounds during Operation Enduring Freedom and Operation Iraqi Freedom.^8^, ^48^ In these war‐wounded patients, TBI, age of less than thirty years, an amputation and multiple extremity injuries with an Injury Severity Score ≥16 are regarded as risk factors for HO development.^8^ Radiographic HO may be apparent 2 weeks after the injury and reaches fully mature in about 3 months^49^ but is not very likely to develop after initial injury period has passed.^8^

The mechanism for traumatic HO remains unclear. It can be considered as a condition of pathological wound healing,^50^ which involves both local and systemic inflammatory reactions.^51^ Studies in the military setting have confirmed a hyper‐inflammatory local and systemic response in patients who developed HO compared with those who did not, associated with elevated Injury Severity Score, bacterial wound colonization and eventual wound failure.^52^, ^53^ It has been proposed that HO development is the result of the induction of progenitor cells by the imbalance in local and systemic factors following traumatic injury to undergo osteogenic differentiation.^54^ However, the injury‐induced osteogenic factors, either local or systemic, have not yet been identified.

**FIGURE 1:**
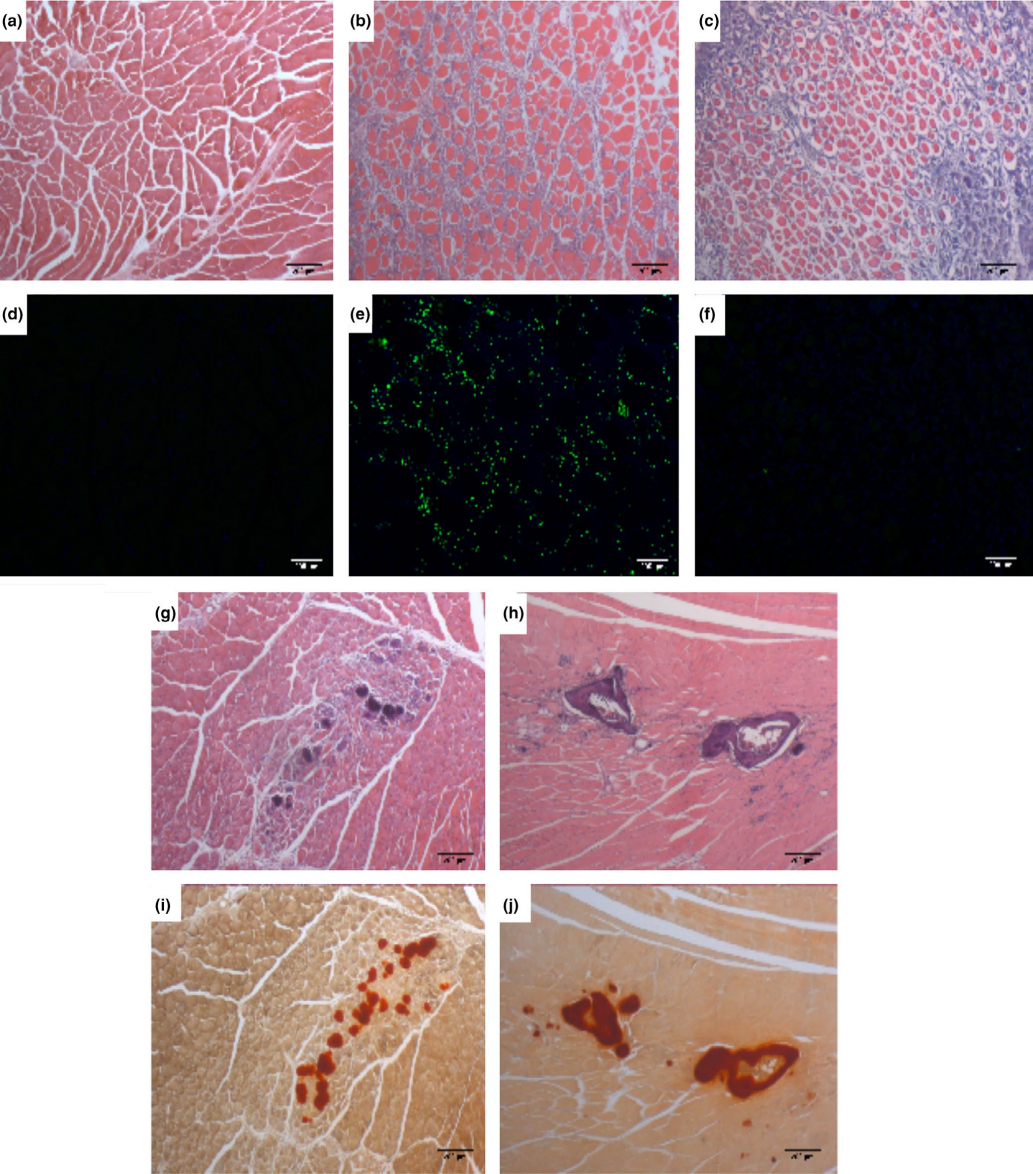
BMP‐7 expression in mouse blast‐traumatized muscle. (A‐C, G&H) H&E staining illustrating muscle tissue morphology at different time points following blast injury: (A) uninjured muscle tissue; (B) 3 days after injury; (C) 7 days after injury; (G) 14 days after injury; and (H) 28 day after injury. Scale bar = 200 µm (D, E, F) Immunofluorescence staining showing the expression of BMP‐7 (green) at early time points following blast injury: (D) uninjured muscle tissue; (E) 3 days after injury; and (F) 7 days after injury. Scale bar = 200 µm (I&J) Alizarin Red staining showing calcium deposition and mature bone formation: (I) 14 days after injury; and (J) 28 days after injury. Scale bar = 200 µm

Members of the BMP family have received the most attention as potential osteogenic factor(s), given their known osteoinductive potency. Previous studies have shown that BMP signalling is normally active in muscle to maintain muscle mass, suggesting the presence of BMPs under physiological conditions.^55^, ^56^ Studies have also shown that BMP signalling is indispensable for muscle regeneration following injury, suggesting that it also involved in pathological conditions.^57^, ^58^ Our studies using a mouse model have shown that, upon cardiotoxin‐induced muscle injury, locally produced BMP‐7 promotes HO formation.^5^ In human blast‐traumatized muscle tissue obtained during debridement of high‐energy wartime extremity wounds from wounded soldiers who developed HO within one year, the increased production of BMP‐1, BMP‐2 and BMP‐4 has been demonstrated.^59^, ^60^, ^61^ We have also shown that, in a preliminary study using a novel mouse blast trauma‐induced HO model, BMP‐7 expression is up‐regulated at the early time points (Figure 1). These findings suggest that BMPs could be the injury‐induced osteogenic factor(s) in the pathogenesis of traumatic HO. The difference in the presence of BMP subtypes we speculate may be due to different injury pathways and harvest times, as well as differences between human and mouse. In addition, others have found that modulation of Smad1/5/8 phosphorylation, with either small molecule inhibitor targeting BMP type I receptor kinase activity or drugs that promote ATP hydrolysis, could mitigate traumatic HO formation resulting from Achilles tenotomy.^62^ Also, knocking out BMP type I receptors (ALK2 and ALK3), as well as using BMP ligand trap A3Fc, significantly reduced HO formation following Achilles tenotomy.^50^ Taken together, these loss of function studies indicate that BMP signalling is critical in traumatic HO and that BMPs represent the most popular candidate injury‐induced osteogenic factor(s) in traumatic HO.

## 5 A UNIVERSAL MECHANISM FOR ACQUIRED HO

**FIGURE 2:**
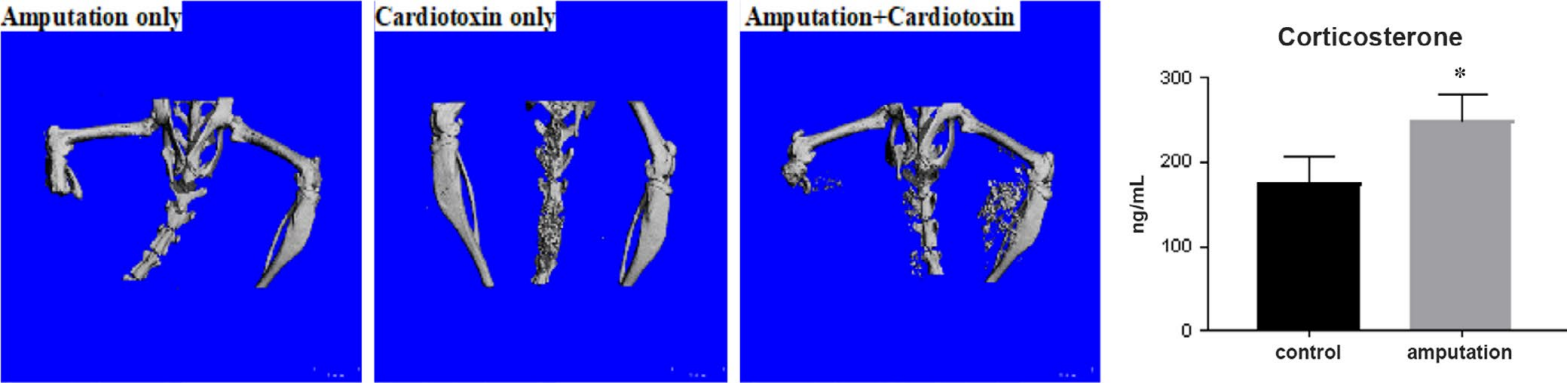
Increased glucocorticoid level promotes ectopic mineralization. (Left) Representative microCT images showing ectopic mineralization in the combined blunt amputation and cardiotoxin injection group. (Right) At 4 weeks after amputation, endogenous plasma corticosterone level remains higher in amputated animals compared with unamputated control animals. (n = 4)

It is noteworthy that decreased plasmin level has recently been associated with HO formation.^63^, ^64^ In these studies, mice deficient for plasminogen gene (congenital plasminogen knockout mice) and mice lacking plasmin activity (transient knockdown of plasminogen by antisense oligonucleotide) both developed HO upon muscle injury, whereas mice with normal plasmin level did not. Down‐regulating the primary inhibitor of plasmin with antisense oligonucleotide targeting α2‐antiplasmin treatment reduced HO volume in heterozygous plasminogen deficiency mice by restoring plasmin activity. Thus, the authors claimed that plasmin prevents ectopic bone formation after muscle injury and that perturbation in the fibrinolytic system could be the underlying mechanism for acquired HO, since hypofibrinolysis is a common occurrence upon CNS and traumatic injury.^64^ In agreement with these studies, we have further demonstrated that glucocorticoids played a role as an upstream regulator of the fibrinolytic system in facilitating HO formation.^65^ Indeed, acquired HO happens most frequently in severe trauma patients when the body is under great stress, which coincides with elevated endogenous glucocorticoid production.^66^, ^67^ The secretion level of glucocorticoids can also reflect the severity of injury^68^, ^69^ and elevated Injury Severity Score is a known risk factor for HO.^8^ Our postulate of glucocorticoid‐induced HO formation is consistent with the observation that the highest rate of HO is found in blast injury, as the massive injuries sustained in blast trauma are seldom seen in the civilian situation.^70^ Our postulate also supports the observation that there is a hyper‐inflammatory local and systemic response in patients who developed HO, because inflammatory cytokines stimulate glucocorticoid production, which normally functions to suppress the body's immune responses being overactivated in life‐threatening situations.^67^ To test our hypothesis, using a model more physiological relevant than the previous glucocorticoid injection model, blunt amputation was made to one leg of the animal to induce a stress response. Preliminary results have shown that, with elevated endogenous glucocorticoid level, cardiotoxin injection to the other leg of the animal could easily result in ectopic mineralization (Figure 2). Taken together, our findings suggest high glucocorticoid level is a key determinant in HO formation. In addition, our glucocorticoid‐induced HO formation theory explains the participation of peripheral nerve system in HO formation independent of CNS injury, for peripheral denervation has been shown to increase the number of glucocorticoid receptor, thus increasing glucocorticoid sensitivity,^71^ as well as plasma glucocorticoid level.^72^ In this manner, peripheral denervation leads to an increased rate of HO.^31^, ^32^

## 6 SYSTEMIC OSTEOGENIC PROTECTIVE FACTOR FOR ACQUIRED HO

**FIGURE 3:**
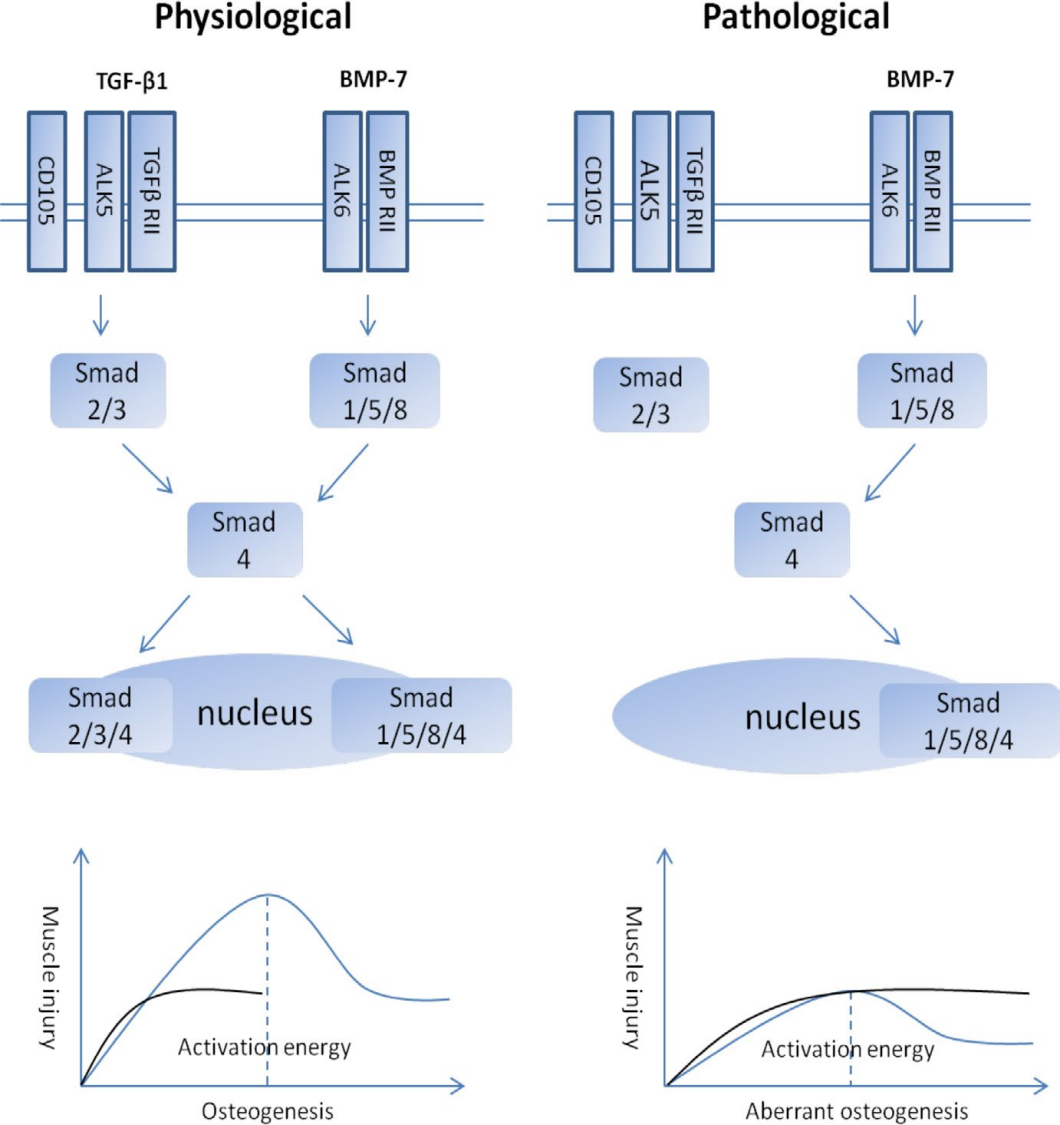
TGF‐β signalling antagonizes BMP signalling by competing for downstream co‐Smad4 for nuclear signal transduction. Under normal physiological conditions, circulating TGF‐β1 acts to maintain the osteoblastic differentiation threshold to control aberrant osteogenesis, whereas under pathological conditions resulting from severe trauma, the level or activity of TGF‐β1 is reduced or blunted, resulting in aberrant osteogenesis

It is interesting that all types of HO occur with a primary defect together with a unifying feature of soft tissue injury. It is well known that a critical threshold of BMP signalling (or ‘activation energy’ of differentiation) is required for osteoblastic differentiation of the progenitor cells for HO formation.^16^ In genetic HO, constitutive activation of BMP signalling lowered this threshold, so that mild injury can trigger or mediate active episodes of bone formation. In the case of acquired HO, when there is no dysregulated BMP signalling, another notion has been proposed for HO formation: under pathological conditions, the protective factors that usually prevent HO formation are removed or inhibited, such that the threshold is lowered and muscle injury can easily result in HO formation as progenitor cells are effectively induced to undergo osteoblastic differentiation.^64^, ^73^ Plasmin is found to be a candidate molecule that prevents dystrophic calcification (DC) and subsequent HO.^64^ However, since plasmin is a promiscuous serine protease, the plasmin substrate that is responsible for this protective role needs to be identified. Transforming growth factor‐beta 1 (TGF‐β1) is a well‐known proteolytic target of plasmin, which releases active TGF‐β1 from its latency‐associated peptide.^74^ TGF‐β1 is also a key factor involved in muscle regeneration^75^ and usually plays an antagonistic role with BMPs in the musculoskeletal system.^76^, ^77^ We therefore hypothesize that TGF‐β1 may function as the physiological protective factor that maintains the osteoblastic differentiation threshold to control aberrant osteogenesis following injury. In our recent study, we have found that glucocorticoid treatment results in lowered circulating TGF‐β1 level, and supplementation of recombinant TGF‐β1 markedly reduced the glucocorticoid‐induced ectopic bone volume following muscle injury.^65^ In addition, inhibition of TGF‐β1 signalling with the small molecule TGF‐β receptor inhibitor SB431542 promotes DC formation following muscle injury (Li et al, accepted for publication). Taken together, these findings suggest that TGF‐β1 could be a key systemic osteogenic protective factor that normally prevents HO formation following muscle injury. Increased systemic glucocorticoid level perturbs the fibrinolytic system, resulting in decreased circulating plasmin and TGF‐β1 levels, thus negating the protective role of TGF‐β1 in pathological calcification. Since the differentiation threshold (or ‘activation energy’) is lowered as a result of lowered circulating TGF‐β1 level (Figure 3), muscle injury‐induced local activation of BMP signalling can lead to HO formation.^5^ A corollary of our hypothesis is that any pathological condition associated with decreased circulating TGF‐β1 level may result in pathological calcification and HO formation. It is noteworthy that a recent study has confirmed decreased TGF‐β1 level following peripheral denervation,^72^ which could be a potential mechanism of peripheral denervation‐induced HO.

## 7 MACROPHAGE PHENOTYPE MODULATION AND HO

In vitro studies have shown that TGF‐β1 inhibits stem cell osteogenesis.^76^, ^77^, ^78^ We have also shown that in vitro supplementation of TGF‐β1, at a concentration similar to that in plasma, almost completely blocked BMP‐7‐induced osteogenesis in cultures of MDSCs,^65^ muscle resident stem cells that have been considered as putative HO cell source in vivo.^40^ This finding confirms that TGF‐β1 may play an inhibitory role on MDSC osteogenesis, which supports our systemic osteogenic protective factor/differentiation threshold hypothesis (Figure 3). However, in vivo results on TGF‐β1 are controversial. TGF‐β1 is a highly pleiotropic factor, and when its effects on the immune and other systems are also taken into consideration, its role can be pro‐osteogenic.^79^, ^80^, ^81^, ^82^, ^83^ We speculate that this is due to the different cellular pathways that lead to bone formation, namely endochondral ossification and intramembranous ossification. Endochondral ossification progresses through a cartilaginous precursor stage which requires TGF‐β signalling for chondrogenesis, whereas in cases of intramembranous ossification, TGF‐β signalling always exerts a direct inhibitory effect on stem cell osteogenesis.^77^, ^84^

**FIGURE 4:**
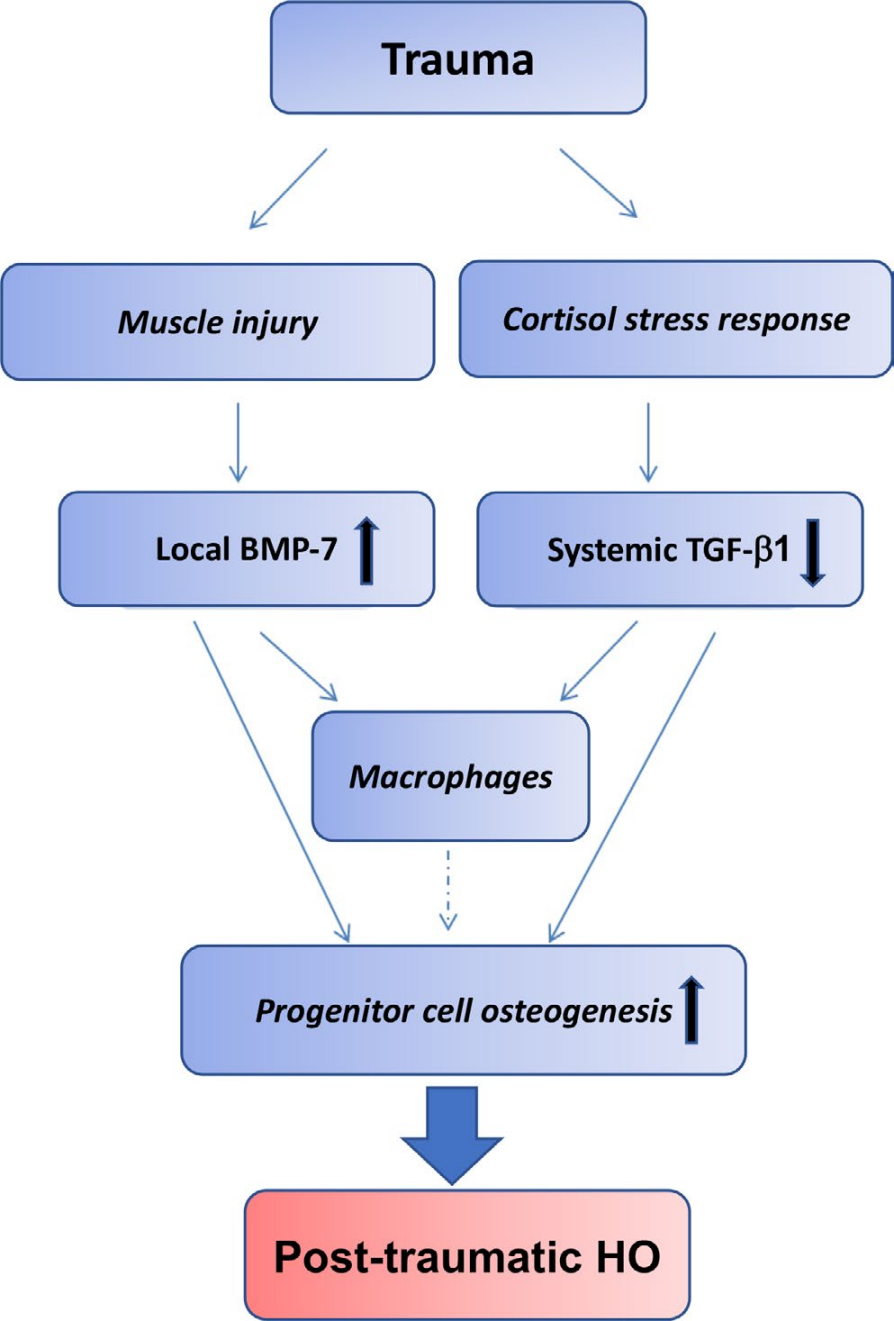
Schematic of a two‐hit model for trauma‐induced HO. Muscle injury‐induced up‐regulation of local BMP‐7 level, and in combination with glucocorticoid excess‐induced down‐regulation of circulating TGF‐β1 level, represents a candidate causative mechanism of post‐traumatic HO formation

We also acknowledge that our postulate may be somewhat simplistic, as there are other cell types that participate in HO formation which could be the direct target of TGF‐β1. Macrophages represent such a target, given that both glucocorticoids and TGF‐β family proteins can modulate their phenotype (Figure 4), which has been reported to affect stem cell osteogenesis.^85^, ^86^ While a number of previous studies have demonstrated that depletion of macrophages prevents HO formation,^20^, ^21^, ^40^ some recent studies have reported that depletion of macrophages or inhibition of macrophage infiltration into damaged tissue can promote HO.^73^, ^87^, ^88^ The reported discrepancy may be due to altered macrophage phenotype resulting from non‐identical clodronate liposome‐based macrophage depletion scheme being used. Indeed, monocyte depletion by clodronate liposome increases local proliferation of macrophage subsets after skeletal muscle injury.^89^ However, few previous studies have characterized the local, tissue level macrophage phenotype after clodronate liposome treatment. Recent studies characterizing infiltrating macrophage phenotype at the HO lesion site showed significant increase in inflammatory Ly6C^hi^ monocyte/macrophages in mice that developed HO compared to mice with only muscle injury.^43^ One study that reported increased HO volume after clodronate liposome treatment showed significant reduction in M2 macrophages, accompanied by a shift in endothelial cell fate towards endochondral ossification.^87^ Another study using a burn injury and Achilles tenotomy HO model confirms that clodronate liposome treatment specifically reduces local Ly6C^low^ macrophage number; however, this time decreased HO volume has been found.^80^ Thus far, no consensus has been reached regarding the relationship between macrophage phenotype and HO pathogenesis. However, it is generally agreed that macrophage heterogeneity exists and that change in macrophage phenotype is a key in the progression and regression of the HO lesion.^80^

Recent studies have shown that specifically knocking out *Tgfb1* gene in macrophage lineage inhibited HO formation in Achilles tendon injury mouse HO models.^79^, ^80^ However, depletion of *Tgfb1* in macrophage did not alter the recruitment of inflammatory cells to the site of injury nor proinflammatory cytokine levels at the tenotomy site, possibly due to the absence of local decrease in TGF‐β1 level.^80^ On the other hand, a trend towards global decrease in serum TGF‐β1 level and plasma cytokine level has been observed.^80^ How this global change in TGF‐β1 level may modulate macrophage phenotype to influence HO pathogenesis remains to be investigated.

## 8 DC AND HO

DC is the deposition of calcium mineral in degenerated tissue, which occurs as a reaction to tissue damage. The majority of DC is normally efficiently removed by phagocytic macrophages, and the damaged tissue repairs fully to its original form.^90^ However, sometimes if tissue repair fails, DC can progress into HO with the participation of connective tissue cells actively laying down a collagenous matrix.^73^ It has recently been proposed that transition from calcification to ossification may be stages of a pathologic continuum, and the progression from DC to HO, associated with an endochondral and intramembranous ossification process, could be the third mechanism leading to HO formation.^91^ Calcification and ossification are best characterized in the cardiovascular system where calcification and ossification often co‐exist in the atherosclerotic lesions,^92^, ^93^ although the mechanisms regulating progression from calcification to ossification in the atherosclerotic lesions are still incompletely understood.^94^, ^95^ The pathogenesis of vascular calcification/ossification actually shares great similarity with muscle DC/HO, in which tissue injury, inflammatory cell infiltration, progenitor cell osteogenic differentiation, etc, are all integral parts of the disease.^96^ Vascular calcification/ossification and muscle DC/HO also share common molecular pathways, that is the same osteogenic factors are present in these two conditions. Interestingly, BMP signalling also promotes vascular calcification,^97^, ^98^ whereas TGF‐β1 protects against the disease progression.^99^, ^100^ Taken together, these data collectively suggest a universal role of BMP and TGF‐β signalling in pathological calcification/ossification.

## 9 SUMMARY

Accumulating evidence suggests a two‐hit mechanism for HO, namely a primary predisposing defect together with soft tissue injury as a second hit to drive disease onset and progression. In genetic HO, the primary defect is gene mutation, whereas in acquired HO we hypothesize that the primary defect results in the removal of the osteogenic protective factors, with circulating TGF‐β1 acting as a key target molecule. In this manner, muscle injury‐induced up‐regulation of local BMP‐7 level combined with glucocorticoid excess‐induced down‐regulation of circulating TGF‐β1 level could be an important causative mechanism of traumatic HO formation (Figure 4).

## CONFLICT OF INTEREST

The authors declare that there is no conflict of interest regarding the publication of this article.
